# The motility-based swim-up technique separates bull sperm based on differences in metabolic rates and tail length

**DOI:** 10.1371/journal.pone.0223576

**Published:** 2019-10-10

**Authors:** Veronika Magdanz, Sergii Boryshpolets, Clara Ridzewski, Barbara Eckel, Klaus Reinhardt

**Affiliations:** 1 Chair of Applied Zoology, TU Dresden, Zellescher Weg, Dresden, Germany; 2 University of South Bohemia in České Budějovice, Faculty of Fisheries and Protection of Waters, South Bohemian Research Center of Aquaculture and Biodiversity of Hydrosensors Zátiší 728/II, Vodňany, Czech Republic; Friedrich-Loeffler-Institute, GERMANY

## Abstract

Swim-up is a sperm purification method that is being used daily in andrology labs around the world as a simple step for *in vitro* sperm selection. This method accumulates the most motile sperm in the upper fraction and leaves sperm with low or no motility in the lower fraction. However, the underlying reasons are not fully understood. In this article, we compare metabolic rate, motility and sperm tail length of bovine sperm cells of the upper and lower fraction. The metabolic assay platform reveals oxygen consumption rates and extracellular acidification rates simultaneously and thereby delivers the metabolic rates in real time. Our study confirms that the upper fraction of bull sperm has not only improved motility compared to the cells in the lower fraction but also shows higher metabolic rates and longer flagella. This pattern was consistent across media of two different levels of viscosity. We conclude that the motility-based separation of the swim-up technique is also reflected in underlying metabolic differences. Metabolic assays could serve as additional or alternative, label-free method to evaluate sperm quality.

## Introduction

Infertility affects 10–15% of couples worldwide, and about 15% of these cases remain unexplained[1,2]. Among various other processes, successful fertilization requires the migration of sperm through the female reproductive tract, a process hard to reconstruct *in vitro*. Except for sperm morphology, current i*n vitro* sperm selection assays in assisted reproduction are mostly based on motility assays, maturity (based on sperm binding to hyaluronic acid), zona pellucida binding, assessment of acrosome reaction, or mucus penetration[3,4]—all processes that require energy.

Sperm cells obtain their energy through two main pathways, oxidative phosphorylation (ATP production through the electron transport chain of the mitochondria) or glycolysis (ATP production in the cytosol by breakdown of sugars). The degree to which these metabolic pathways are employed in sperm is species-specific and either oxidative phosphorylation or glycolysis can be more pronounced under certain conditions[5]. For instance, it is known that in murine sperm, glycolysis is the dominant pathway. In bovine sperm, oxidative phosphorylation seems to be preferred[5–8], but it was also demonstrated that sperm motility can be maintained in the presence of glucose and electron transport chain inhibitors[9]. In human sperm, both pathways are essential for energy production. Again, glycolysis is sufficient to maintain motility if glucose is present in the medium[10,11]. Thus, sperm of many species are predicted to be able to switch between pathways depending on the conditions in the female reproductive tract, substrate and oxygen concentrations[8,12].

As most of the sperm’s energy goes towards motility, motility is commonly used as an indicator for predicting fertilization success[8,13,14]. It has been suggested that higher energetic capacity may be important under the conditions of high sperm competition occurring in birds and external fertilizers[15,16].

**Fig 1:**
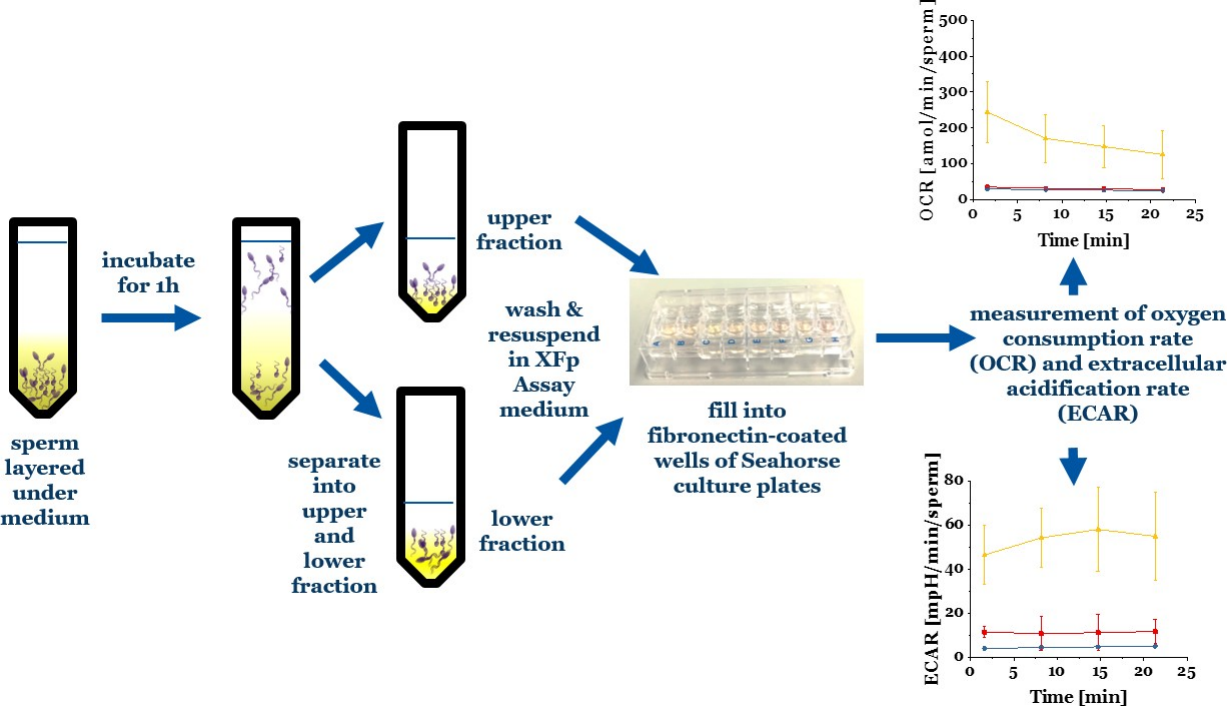
Schematic of the workflow for swim-up of bull sperm and subsequent metabolic measurements.

Swim-up is a simple method routinely used in the clinic to separate motile from non-motile spermatozoa *in vitro*[17] whereby sperm are allowed to swim upwards through an overlaid medium (see schematic workflow in Fig 1). Sperm that do not migrate into the upper medium form the lower fraction. Compared to these sperm, sperm that swim up into the upper medium (upper fraction) display increased motility, higher average velocity, higher percentage of normal morphology and generate improved fertilization rates *in vitro* in mammals[18–23]. Therefore, the swim-up method is a good model for mimicking the requirements for sperm transport in the female reproductive tract to the fertilization site[22]. While it is clear that the upper fraction sperm are enriched to be used in artificial reproduction technologies in humans, the underlying mechanism that promotes their accumulation in the upper fraction seems yet unknown. Do these cells show higher metabolic activity, a different proportion of oxphos and glycolysis or higher and more efficient ATP production and usage? It is not clear to what extent positively selected swim-up sperm with proven increased fertilisation rates *in vitro* will represent successful sperm migration in the female reproductive tract *in vivo*. Under natural conditions, sperm cells migrate through very complex environments in the female reproductive tract, characterized by a wide range of pH, viscosities, surface morphologies. For instance, the swim-up medium is less viscous than the female reproductive tract mucus. During their migration through the reproductive tract, sperm cells encounter a wide range of viscosities, and high viscosity is thought to act as a selective agent or barrier by the female. It is also known that the flagellar beating pattern of sperm is altered in high viscosities.[24] We take this as an incentive to investigate the metabolism of the different sperm swim-up fractions in a medium with viscosity equivalent to the female reproductive tract mucus[25,26]. There is little information on whether differences in motility, and more specifically, response of cells to the swim-up test, are associated with differences in metabolic rate, or metabolic pathways. This seems important because under oxidative, but not glycolytic sperm metabolism, sperm damage by oxygen radicals may occur[27]. This notion may also be important in non-human vertebrates because females of non-human vertebrates can store sperm for days to months, even years[28] and therefore it is not clear how the short-term success identified by swim-up will translate into fertilisation success of sperm that have been stored. For example, if swim-up success would be caused by a high rate of oxidative sperm metabolism, it is conceivable that this high short-term oxidative expenditure may translate into oxidative damage in the mid- or long-term.

Thus, both, clinical analysis as well as sperm selection in assisted reproduction technologies in humans and animals would benefit from information about the underlying metabolic pathway of the swim-up fractions. Here we compare the metabolic activity of sperm in swim-up fractions in 12 bulls from three genetically different breeds. We perform metabolic measurements before and after the swim-up procedure and compare metabolic rates from oxidative phosphorylation and glycolysis of the upper and lower fractions and analyse whether sperm metabolism is similar across two levels of viscosity and so predicts motility through environments of different viscosity.

## Methods

### Schematic of workflow

Fig 1 illustrates how the separation and subsequent metabolic measurements of bull sperm swim-up fractions were performed.

### Preparation of bovine sperm

Cryopreserved bull semen from 12 bulls from three different breeds (4x Holstein, 4x Fleckvieh & 4x Angus) was obtained from Masterrind GmbH. The semen straws (obtained from bull’s ejaculates) were thawed in a 38°C water bath for 2 minutes and diluted in 1 mL modified Tyrode’s medium (SP-TALP). SP-TALP was made from SP-TL (Caisson labs), 100mM sodium pyruvate (Gibco), 50 mg/mL Gentamicin (Caisson labs) and 6 mg/mL bovine serum albumin (fraction V, Sigma) The vial was centrifuged at 300g for 5 minutes in soft centrifugation mode. The pellet was resuspended in SP-TALP and kept in the incubator (37°C) until further use.

### Swim-up

Swim-up was performed immediately by resuspending the washed sperm pellet in 350 μL SP-TALP. The resuspended sperm where then carefully layered under 1 mL SP-TALP in an Eppendorf vial. This vial was placed in the incubator for swim-up for 1 h. Subsequently, the upper 1 mL were withdrawn and placed in a separate vial (see schematic in Fig 1). Upper and lower fraction of the swim-up were washed once with SP-TALP at 300g for 5 minutes.

### Motility assays

The sperm video recording was performed on three bulls immediately after the swim-up and before the metabolic measurements. Different sperm fractions were placed on a glass slide under the microscope (Leica DMI inverted microscope) with heating stage with a HT50 control unit (Minitübe) (temperature was set at 38°C). All spermatozoa in the observation field (50–300) were recorded for at least 5 sec with a PCC software and Phantom Highspeed camera at 200 fps, using 10x objective magnification and phase contrast condenser. Video records were stored in AVI format prior to analysis. Motility analysis was done with the CASA plugin of ImageJ[29] modified according to Purchase & Earle (2012)[30] delivering overall motility, average path and straight line velocity (S1 Fig).

### Seahorse metabolic assays

Metabolic measurements were performed on semen samples from 12 bulls with a Seahorse XFp device from Agilent with eight well cell culture plates. One day before the assay, the sensor cartridges were hydrated at 37°C in calibration solution (Agilent). On the day of the assay, each well bottom of the cell culture plates was coated with 10 μL fibronectin (1 mg/mL, Sigma F1141) and incubated until dry to allow sperm attachment to the well bottoms (S2 Fig). The washed sperm pellet was resuspended in XFp Assay medium (XF Base medium, sodium pyruvate, glutamine, glucose, pH 7.4, sterile filtered) to obtain a cell concentration of 10^5^−10^6^ per well. 50 μL of cell suspension was added to each of six wells (two control wells without cells) and centrifuged for 1 minute at 600 rpm to ensure cell attachment. Assay medium was added to the wells to a total of 180 μL per well. The cell culture plates were kept in the incubator for 10 minutes to acclimatise. Then, the metabolic measurements with Seahorse (Agilent) were started by calibrating the sensor cartridge in the calibration solution. After the calibration, the cell culture plate was inserted. During the Seahorse measurements, the temperature was kept at 37°C and automatic injections were programmed as desired. Oligomycin A injection was performed in order to measure the ATP production due to oxphos. Oligomycin A blocks the proton channel of the ATP synthase, an enzyme necessary for oxidative phosphorylation of ADP to ATP[31]. Thus, the energy production by oxidative phosphorylation is inhibited after injecting Oligomycin A to the bull sperm fractions in the Seahorse device. 20 μL of 10 μM Oligomycin A was injected to each well after basal rate measurement, so that a final concentration of 1 μM Oligomycin A was reached in each well. The ATP production due to oxphos can be calculated by subtracting the OCR rate after Oligomycin injection from the basal OCR rate.

After the metabolic measurements, the viability of cells was checked by viability stain (Live/Dead Sperm viability kit (ThermoFisher L7011, S2 Fig in supporting information) and sperm cells from each well were released by vigorous pipetting and counted in a Neubauer counting chamber in order to normalize the values to sperm numbers.

For the investigation of metabolic rates of bull sperm in two different levels of viscosity, swim-up of bull sperm was performed in sperm medium, as described earlier, then, the different sperm fractions were subsequently immersed in highly viscous medium (by supplementation of 1% methyl cellulose, Serva, Feinbiochemica Heidelberg, 350–550 cP at 2% in H_2_O at 25°C). Methyl cellulose is an inert, biocompatible natural polymer that has been used in various studies to mimic the viscosity of body fluids, also for studying sperm motion[25,26,32–34]. Its viscosity is about 100 times higher than conventional sperm medium (at low shear rate of 5 s^-1^), similar to female reproductive tract mucus. Eamer et al. studied sperm motion in dependence of viscosity of medium by preparing different methyl cellulose solutions. Their data show that with increasing viscosity, the average sperm velocity decreases while the sperm linearity increases[33]. Furthermore, Gonzalez-Abreu et al.[26] reveal a beneficial effect of raising viscosity of the media on the sperm parameters in terms of higher proportion of fast linear spermatozoa and lower percentage of spermatozoa with slow non-linear movement.

### Luminescence-based ATP kit

Total ATP content of sperm was measured on semen samples from 12 bulls with the luminescence-based ATPlite kit (Perkin Elmer). First, an ATP standard curve was set up by preparing an ATP dilution series from 10^−5^ M ATP down to blank. The ATP content of sperm was measured by pipetting 100 μL sperm cell solution into each well of a black 96-well plate, adjusted to 37°C. 50 μL of lysis solution were added and the plate was shaken for 5 minutes. 50 μL substrate solution (luciferase/luciferin) were added to each well. After shaking for 5 minutes, the plate was dark adapted for 10 minutes and subsequently luminescence was measured with a plate reader (Infinite 200 Pro, Tecan) at 37°C.

### Statistical treatment of data

Data were analysed using R version 3.5.2 (2018-12-20)[35] and the lme4[36] and glmmADMB package[37,38]. The data on OCR, ECAR, ATP production and ATP content were binomially distributed and skewed. We modeled these response variables with a negative binomial fit. The ratio of OCR/ECAR was modeled with a binomial fit, using the *cbind* function. We built generalised linear mixed models (GLMM) testing for the effect of the factors viscosity of the medium (low vs high) and fraction (upper vs lower) on OCR, ECAR, OCR/ECAR ratio, ATP production, and for the effect of fraction on the ATP content of sperm (S1, S2, S3, S4 and S5 Tables). For repeated measures per bull, bull ID was added as a random factor. Initial models contained the interaction of the factors fraction and viscosity, or, in the case of ATP content, fraction, and the random factor bull ID. We reduced the full models stepwise backwards and compared the final models to their respective null models to select the best model using the *anova* function. The final models are presented in the supporting information.

Motility data and tail length were modeled with a Gaussian fit. We tested for the effect of the factor sperm fraction (upper vs. lower) in linear mixed models (S6 and S7 Tables). Again, bull ID was added as a random factor to account for repeated measures per individual.

## Results and discussion

### Metabolic rates of bovine sperm after swim-up

**Fig 2:**
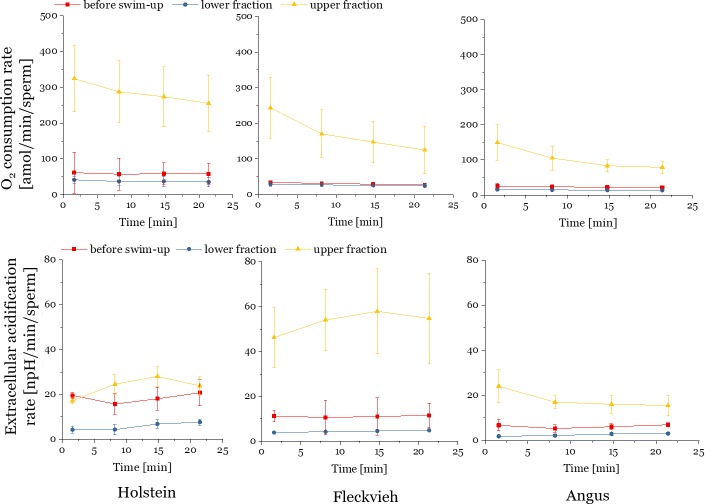
Basal sperm metabolic rates over time in three individuals belonging to different breeds. Oxygen consumption rates (OCR) (top) and extracellular acidification rates (bottom) of bull sperm before swim-up (red symbols), upper (yellow symbols) and lower fractions (blue symbols) after swim-up procedure of three bulls of different cattle breeds. Each data point is obtained from a mean value from 3 wells and the data were normalized to the number of cells in each well. Error bars are standard deviation from 15 reads and 3 wells.

The rate of oxidative phosphorylation and glycolytic rate are obtained by simultaneously measuring the oxygen consumption rate (OCR) and extracellular acidification rate (ECAR) in an extracellular flux analyzer. The oxygen consumption rate is a direct measure of the mitochondrial electron transport rate and thereby stands as an equivalent for oxidative phosphorylation, during which oxygen is consumed by the cells. The extracellular acidification derives from the lactic acid formed during glycolysis. For a previous application in sperm see Tourmente et al.[6]. The basal rates for OCR and ECAR of the upper, lower and before swim-up fractions were measured for 22 minutes comprising four measurement points each consisting of 15 data points (Fig 2).

The upper fraction showed higher OCR across three bulls from three different breeds (Holstein, Fleckvieh, Angus) (Fig 2, yellow data points). The average OCR of upper fraction sperm was up to an order of magnitude higher than in the “before swim-up” and lower fraction sperm population (Fig 2).

Generally, the low ECAR values of all sperm fractions indicate that bull sperm perform little glycolysis. The three bulls differed in the temporal change in glycolytic activity (Fig 2, lower panel). The upper fraction (yellow points, Fig 2) usually showed higher ECAR activity compared to the “before swim-up” sperm (red points, Fig 2) and consistently higher activity compared to the lower swim-up fractions (blue data points, Fig 2).

This indicates that the swim-up selects sperm with higher metabolic rates. The fact that the upper fractions display higher OCR and ECAR was not caused by larger sperm numbers in the lower fraction, because all OCR and ECAR values were normalized to the sperm number per well. The density of sperm does not influence their metabolic activity[39]. On average, the before swim-up fraction contained 4x10^6^ cells/mL, the lower fraction 2x10^7^ cells /mL and the upper fraction 8x10^5^ cells/mL, which means that 20% of cells from the initial sample (before swim-up) migrate to the upper fraction during the swim-up. A low proportion of motile sperm remaining in the lower fraction may cause the OCR in the lower fraction to show lower average values. We tested for this idea in sperm samples from three bulls by normalizing OCR and ECAR to the number of motile sperm in each fraction (S3 Fig) even though such normalization has the caveat that metabolically active but non-motile sperm are excluded.

Motility was measured immediately before the metabolic measurements (see Methods). The swim-up accumulates the motile sperm in the upper fraction (motility values of 45–65% in the upper fraction and the non-motile sperm (motility 12.5–32%) in the lower fraction (S3 Fig, part a). Standardising OCR values to the number of motile cells shows the differences between “before swim-up” and “upper fractions” are now diminished in two individuals and retained in one (S3 Fig, part b). Therefore, the OCR separation by swim-up is partly but not exclusively related to motility.

Normalizing ECAR in the same way shows a large range of ECAR per motile sperm in the Holstein bull before swim-up (S3 Fig, part b, right graph) (20–160 npH/min/motile sperm), reduced in the upper (40–74 npH/min/motile sperm) and lower fraction (10–32 npH/min/motile sperm). In the Fleckvieh bull, the upper fraction shows by far higher values than the before swim-up and lower fraction. In the Angus bull, all fractions display relatively low ECAR values. This illustrates that the swim-up does not generally select sperm with higher glycolytic rate (only in Fleckvieh was this the case).

Plotting the average basal OCR over the average basal ECAR (see S4 Fig) results in the characterisation of the metabolic phenotype, or energetic potential, i.e. the ability of the cells to change the metabolic pathway and respond to changes in the energy demand. Analysing the metabolic phenotype of the upper and lower fraction (S4 Fig) suggests that the upper fraction sperm had more energetic potential, as indicated by the upper fraction moving more to the upper right.

Regardless of the actual values, the ratio of OCR/ECAR can indicate a relative preference of metabolism for either oxidative phosphorylation or glycolysis[6,40]. The upper fractions show a OCR/ECAR ratio mean value more towards glycolysis than the “before swim-up” and lower fractions (S5 Fig) (p<0.0001, see supporting information), indicating a shift in metabolic pathway usage occurs. This difference in OCR/ECAR ratio was consistent across both levels of viscosity (p<0.043, see supporting information). The OCR/ECAR ratio does not represent overall metabolic activity, but rather shows a shift in metabolic pathway usage.

### The influence of medium viscosity

**Fig 3:**
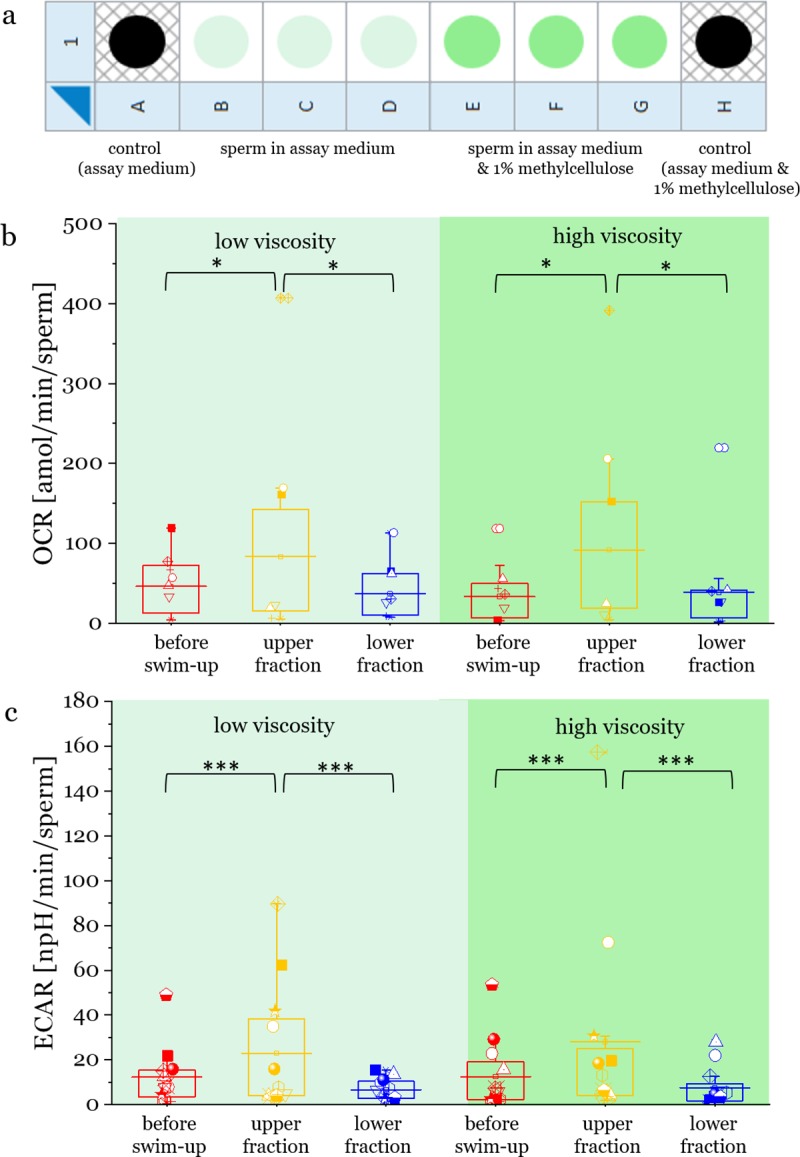
**Oxygen consumption rate (OCR) in low viscosity medium (Assay Medium, indicated as light green) and high viscosity medium (Assay medium supplemented with 1% MC, indicated with darker green) of bull sperm after undergoing the swim-up procedure in low viscosity sperm medium SP-TALP.** a) Schematic of the sample distribution in the 8 well plate. This triple-well measurement was performed on each fraction (upper, lower and before swim-up) and two control wells with the two levels of viscosity were used as background level. b) Oxygen consumption rate (OCR) and c) extracellular acidification rate (ECAR) for the before swim-up sperm, the upper and lower swim-up fractions of 12 bulls of three different breeds (Holstein, Fleckvieh, Angus) in two levels viscosity (labelled with light and dark green background). The horizontal lines across the boxes display the median values. The upper fraction shows significantly higher OCR (OCR, p = 0.02) and ECAR (p = 0.00019) compared to the lower fraction and before swim-up. Viscosity does not change the OCR or ECAR values significantly.

So far, we have analysed metabolic parameters in standard medium. The question is, if the separation by swim-up will result in similar separation effects in a different environmental condition, e.g. different viscosity. Subsequent to swim-up, we immersed the different fractions into high and low viscosity medium and tested their metabolic performance in the extracellular flux analyzer. If the results are similar, it would prove that swim-up separates fractions that are consistent in their performance across different viscosities. We measured the metabolic rates of the different fractions in two different viscosities (see setup Fig 3A). Adding 1% methyl cellulose to a conventional assay medium results in viscosity values several orders of magnitude higher (at shear rate 5 s^-1^)[25,26]. This viscosity mimics that of fluids of the reproductive tract, reported to be 0.1–1.0 Pa s [41] and has previously been used to study sperm motion in viscous environment similar to the *in vivo* conditions[32–34,42].

Across both viscosities, sperm with higher OCR (Fig 3B) and ECAR (Fig 3C) accumulated in the upper fractions. Note that the results in standard medium confirm our earlier measurements. There were no differences in sperm OCR rates between low and high viscosity. This indicates that swim-up collects sperm in the upper fraction that display a higher respiratory and glycolytic activity and their overall metabolic rate allows them to penetrate fluids of different levels of viscosity.

The difference in energetic phenotype between upper and lower fraction was also maintained through both levels of viscosity, as was the difference in OCR/ECAR ratio (p<0.043, see S5 Fig).

### Swim-up selected sperm show higher ATP production by oxphos

**Fig 4:**
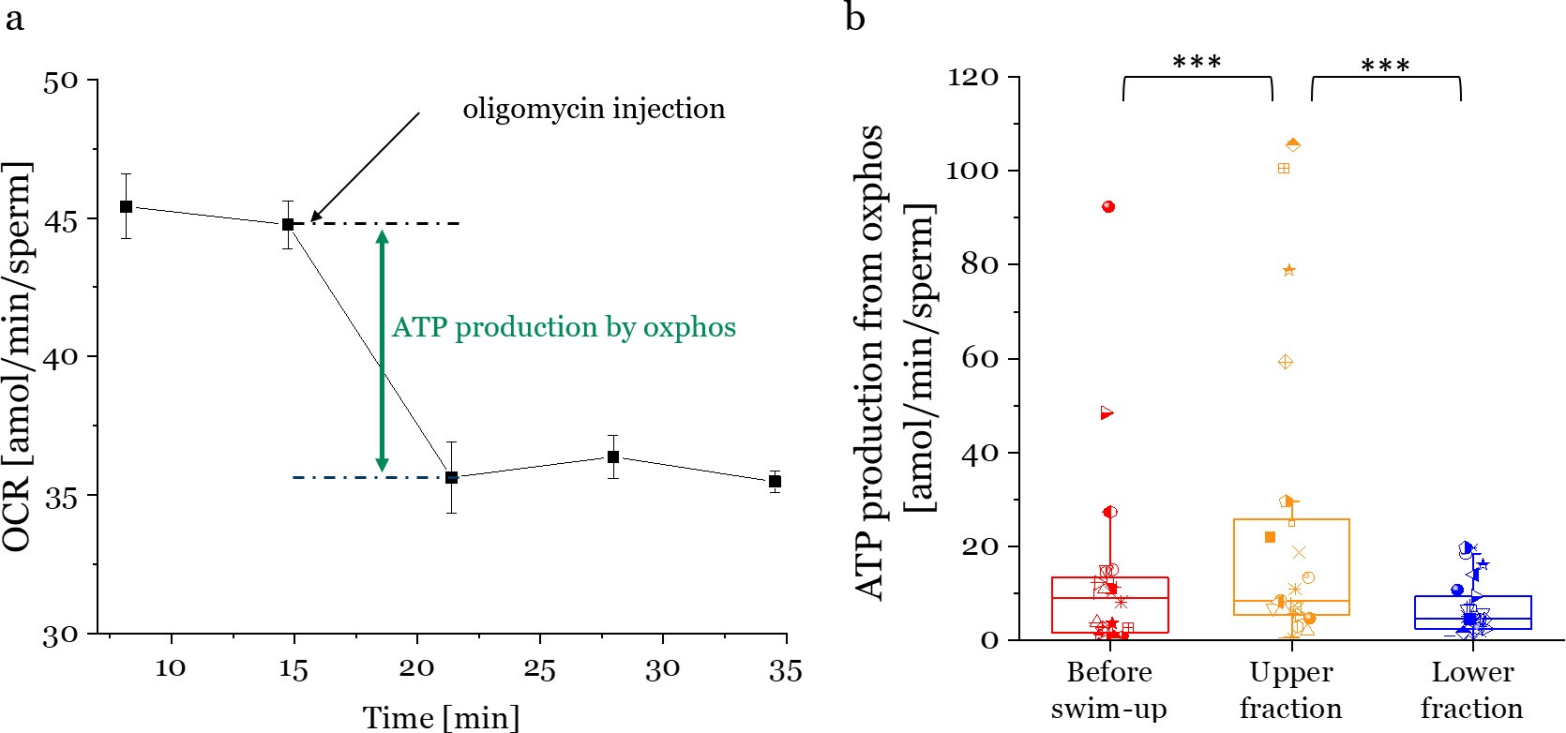
ATP production by oxidative phosphorylation of different swim-up fractions. a) ATP production from oxphos is calculated as the difference between basal OCR rate and OCR rate after oligomycin injection. b) Average ATP production due to oxphos of unselected sperm (before-swim-up), upper and lower swim-up fractions. Each box contains data from 12 bulls. Horizontal lines through each box are the median values, lower and upper limits are 25^th^ and 75^th^ percentile, respectively.

A crucial question is to what degree metabolic differences translate into differences in ATP production. Inhibiting the electron transport chain with Oligomycin A allows a calculation of ATP production rate by oxidative phosphorylation (Fig 4A). The lower fractions (Fig 4B) produced less ATP by oxidative phosphorylation than the upper fractions (p = 0.0001, see S4 Table in SI). The upper fractions also had higher values than the initial sperm sample (before swim-up). ATP production was similar in higher compared to lower viscosity for all fractions (S6 Fig in supporting information).

These results indicate that the swim-up separates sperm into fractions of higher and lower ATP production, but that swim-up does not reflect a precise measure of ATP production.

### Higher metabolic and kinetic activity leads to reduced ATP content

**Fig 5:**
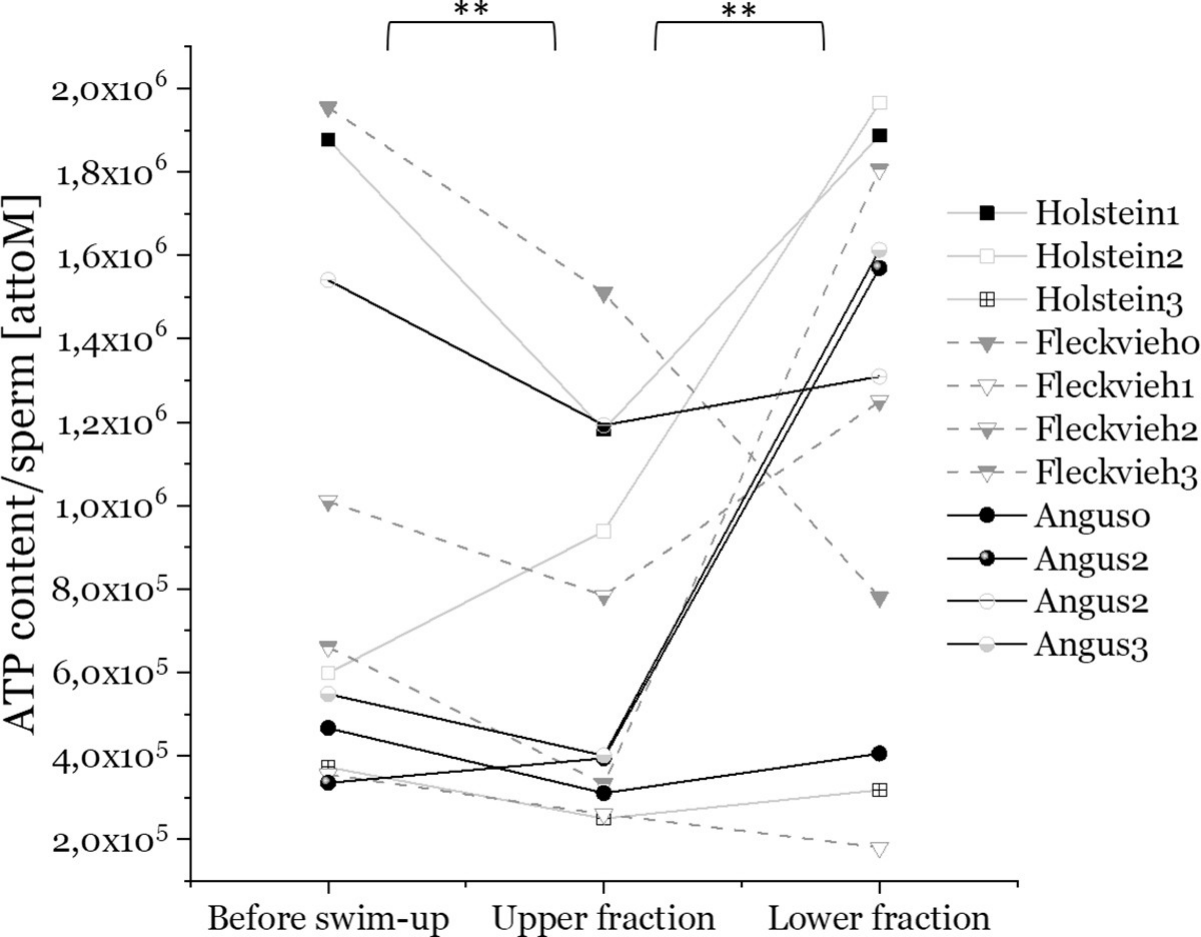
Total ATP content per sperm cell of before swim-up fractions, upper and lower swim-up fractions from 12 bulls measured with a luminescence-based ATP kit. Each condition was measured three times.

Next, we looked at the current ATP content of upper and lower fraction sperm, determined in 11 bulls using a luciferase-based luminescence kit (Fig 5). The bulls showed lower total ATP content in their upper fractions compared to the before swim-up sperm and the lower fraction (p = 0.01, see S5 Table). The fact that increased metabolic rates in the upper fraction (Fig 2 and Fig 3) are associated with lower total ATP content (Fig 5) suggests that upper fractions show increased ATP expenditure, perhaps caused by the metabolic activity during swim-up.

**Fig 6:**
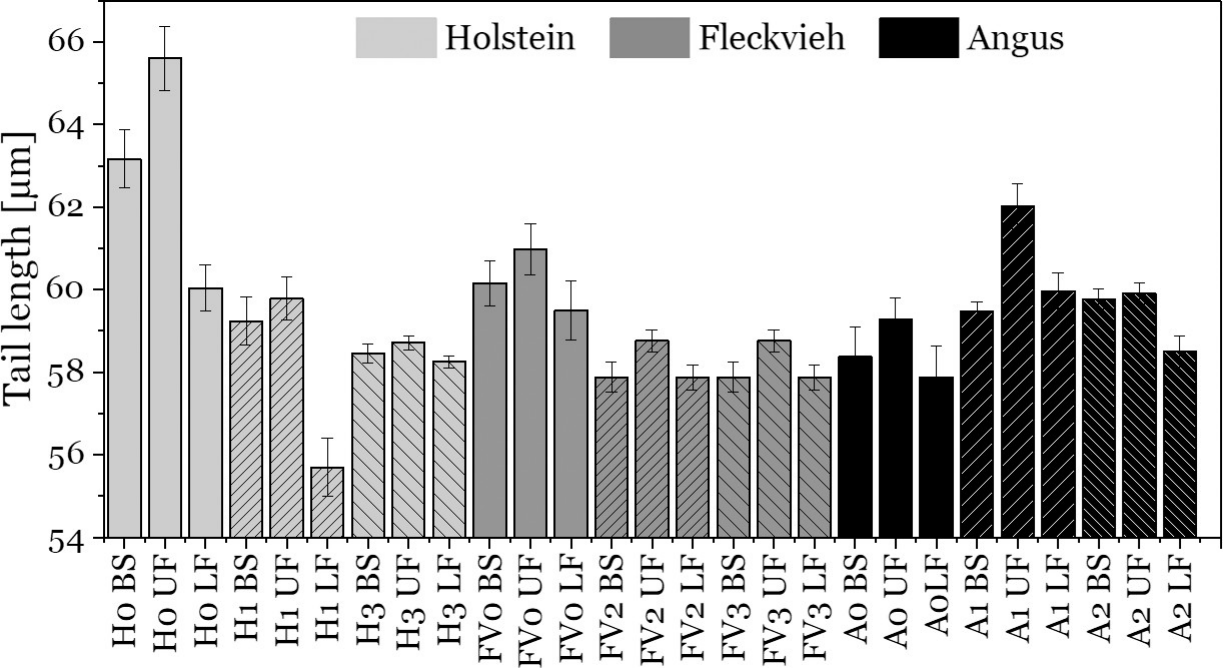
Sperm flagella length of swim-up fractions from 9 bulls of different breeds. 100 sperm cells were measured for each bar. Error bars are standard error of the mean. Upper fractions contain consistently longer sperm tails (estimate = 1.05, p = 0.04) compared to the initial sperm sample. The lower fractions contain consistently shorter tails (estimate = -0.98, p = 0.05). BS = before swim-up. UF = upper fraction. LF = lower fraction. H0-3 = Holstein bulls, FV0-3 = Fleckvieh bulls, A0-2 = Angus bulls.

Theory suggests that power output scales with the cube of sperm tail length and can thus be considered a selection parameter for more efficient sperm swimming[43]. Indeed, swim-up selected sperm with longer flagella in at least one study[22]. Reasons for the selection of longer sperm during swim-up might be on the one side that longer flagella contain more molecular motors inside their axoneme and are therefore able to perform stronger power strokes. On the other side, longer flagella might have longer midpieces and thus a larger mitochondrial sheeth wrapped around them. This would explain a higher energy production by mitochondrial respiration. In our study (Fig 6) we found in all individuals that the upper fraction of swim-up sperm contained longer sperm tails compared to the initial sperm sample (p = 0.04). The lower fraction consistently contained shorter tailed sperm (p = 0.05, Fig 6 and S7 Table). The average length difference was 1.04μm, which is similar to the previous study on bull sperm that showed an average length difference of 0.62μm[22].

## Conclusions

This study examines metabolic differences underlying the swim-up method used for sperm analysis in clinical and veterinary settings. We confirm that swim-up selects bull sperm of higher motility. We additionally reveal that swim-up as a standard method for preparing cryopreserved sperm separates the cells into fractions of high and low metabolic rates and these are based on both, higher oxygen consumption rates and extracellular acidification rates. We also show that swim-up separates sperm of high and low ATP production but that upper-fraction sperm cells have lower ATP content and therefore possibly have higher ATP expenditure. We combine the swim-up method with subsequently immersing the sperm in high viscosity medium and show that the level of viscosity does not significantly change their metabolic rates. We confirm a previous notion that larger sperm are selected[22] and show that the selected fraction differs not only in motility, but also in metabolic rate and ATP production., Overall, this investigation provides a metabolic explanation of why the swim-up method selects sperm that seemingly are functionally superior, an insight that may be useful in animal reproduction science. This notion could extend to the sperm of other animals and humans.

The upper swim-up fractions display both highest oxygen consumption rates and highest extracellular acidification rates. Whether swim-up selects sperm with highest oxygen consumption rates or the highest extracellular acidification rates remains an open question. Our results showing that swim-up selects sperm in the upper fraction with highest ATP production from oxphos may suggest that oxphos plays a key role, but the causal connection between the two remains open.

Our data are consistent with previous studies using different methods in showing a low glycolytic contribution to bull sperm metabolism[5,8] and we, therefore assume our study to be confirmative to previous results. Whilst the low glycolytic activity argues for a substantial utilisation of oxphos, glycolysis is also discussed as being essential to supply energy to sperm regions far from mitochondria to support motility[7]. For example, inhibiting oxphos while stimulating glycolysis maintains full sperm motility[44]. Other studies suggest that diffusion of ATP in sperm flagella is enough, if there is enough ATP stored in the tail, as it is the case in bull sperm[45]. Additionally, it was observed that sperm cells, especially bovine sperm cells, continue being motile in medium without glucose for a long time, suggesting that gluconeogenesis might play a role in energy supply[46]. Yet, clear evidence is lacking.

### Viscosity and its effect on energy expenditure and oxidative damage

Sperm cells in the upper fraction are more likely to penetrate fluids of different levels of viscosity. This supports the hypothesis from simulation-based studies that suggest that sperm cells swim more efficiently in highly viscous media by adjusting their flagellar waveform[47]. In this study, it is predicted that, by decreased cell yawing and reduced wavelength, the sperm cell increases its power output in higher viscosity by a factor of four. The change in flagellar waveform induces a greater efficacy (velocity per mechanical power) of the sperm motion, thereby reducing the need for increased energy expenditure. Our study also reveals that increasing the viscosity of the medium does not improve the separation power of sperm cohorts based on metabolic differences, because no differences in metabolic rates are observed in these settings. We, therefore, recommend that higher viscosity should not be used to separate sperm; among others this is to avoid the possibility of the Brynhild effect[48]. This effect applies if separation or quality control thresholds for sperm are set too high, in either the female or in artificial settings, sperm may actually incur reduced functionality or higher energy expenditure (as our data suggest) and not be useful for fertilisation. For example, a threshold that requires large energy expenditure may select for sperm utilising oxphos, which results in 15 times more ATP compared to glycolysis. However, oxphos, responsible for ~90% of a cell’s ROS production[49], may increase ROS damage of cell membranes and DNA[49] thereby reducing sperm function and disabling the threshold[50]. Future work should test to what extent upper fraction sperm accumulate oxidative damage.

Longer migration distances of sperm (e.g. from the anterior vagina in case of natural mating) have been suggested to be one way of quality control for sperm[51]. While it has been argued that such long distances make high metabolic rates beneficial (the winning-the-race argument[51]) it seems that because sperm are stored by females before fertilisation, low metabolic rates may be beneficial because ATP would be used up slower and sperm retain motility for longer. It was predicted that ATP content could be an interesting parameter coupled to fertility and gives supplementary information about sperm quality.[52] It was reported that ATP content does not necessarily correlate with motility, but is influenced by preservation method and post-thaw incubation time. Yet, further research is needed to understand the connection between ATP content and fertility. In our example, we find that low ATP reserves likely indicate high ATP expenditure. Combining measures of ATP reserves and ATP production rate might be a useful prediction parameter for sperm quality.

In our study we include 12 bulls from three genetically different breeds. The variation in the investigated parameters (OCR, ECAR, ATP content, ATP production, tail length) between bulls is large and therefore prevented an analysis of breed-specific traits. Whilst not the focus of the work presented here, breed-specific sperm metabolism may be an interesting aspect of predicting fertility.

Since the swim-up procedure is a common sperm preparation method and a model to mimic sperm migration we think it might be reasonable to test the metabolic differences between upper and lower sperm fractions in human sperm for its potential clinical applicability, especially given that motility and metabolism are not perfectly correlated. An interesting extension would be to assess sperm metabolism in patients with low or no sperm motility or abnormal morphology because these impede the standard motility assays. These sperm would be evaluated as “infertile” with the standard spermiogram, but might still be able to fertilize under certain conditions. Also, immotile sperm or sperm with abnormal morphology which impairs motility, might still show metabolic activity. Further prospects of this method lie in the assessment of metabolism in real time in sperm exposed to drugs, medium additives or toxins.

In conclusion, we propose that measuring metabolic activity of sperm can be an important indicator for sperm quality and their migration success.

Metabolic analysis, in addition to the conventional motility analysis, will give a more comprehensive picture of sperm quality, also predicting fertilization success in vivo. Moreover, metabolic analysis is also particularly useful in cases of asthenozoospermia and teratospermia, where the standard motility assays fail to evaluate sperm quality. Furthermore, metabolic measurements of sperm cells can reveal differences in the usage of or switching between certain metabolic pathways in different environments. Metabolic potential, ATP production and ATP content are important parameters when looking at environmental conditions and their effect on sperm quality and migration success, not only in a momentary analysis (as is CASA), but a method that can predict sperm migration success with a more holistic approach.

## Supporting information

S1 Fig **Average path velocity VAP (top) and straight line velocity VSL (bottom) of different bull sperm swim-up fractions** in low viscosity (left) and high viscosity (right) for three different bulls. N >600 sperm cells for each bar. Videos of 10 second length were recorded and then analyzed in one second increments. For each condition and sperm fraction, 3 videos were recorded. Error bars show standard error of the mean.
(TIF)

S2 Fig **Sperm cell attachment** (left image) during metabolic measurement in the XFp Analyzer and viability stain (right image) after seahorse measurement shows viable sperm cells under assay conditions (37°C, assay medium supplemented with glutamine and glucose).
(TIF)

S3 Fig Metabolic rates normalized to overall motility. a) Overall motility of swim-up fractions of bull sperm. b) OCR (left) and ECAR (right) normalized to number of motile cells in each fraction. N≥4 replicate measurements.
(TIF)

S4 Fig The metabolic potential is displayed by plotting oxygen consumption rate (OCR) over extracellular acidification rate (ECAR) for the upper and lower swim-up fractions of 12 bulls from 3 different breeds. (TIF)

S5 Fig OCR/ECAR ratios of the sperm fractions before swim-up, upper and lower swim-up fractions. Each box displays OCR/ECAR ratios of 12 bulls, the horizontal line across each box is the median value.
(TIF)

S6 Fig **ATP production** from oxphos calculated from oligomycin injections (see Fig 6A) of swim-up fractions in low viscosity (left panel) and high viscosity (right panel). Each box was obtained from 12 semen samples from different bulls. Horizontal lines through each box are the median values. Viscosity does not influence the ATP production significantly (p = 0.07). The ATP production in the upper fraction is significantly higher (p = 0.0001) than in the lower fraction and before swim-up.
(TIF)

S1 Table Generalised linear mixed model (OCR ~ fraction+(1|ID), family =“nbinom”). OCR = response variable, fraction = factor, (1|ID) = random factor, SE = standard error.
(TIF)

S2 Table Generalised linear mixed model (ECAR ~ fraction+(1|ID), family =“nbinom”), ECAR = response variable, fraction = factor, (1|ID) = random factor, SE = standard error. (TIF)

S3 Table Generalised linear mixed model (cbind(OCR,ECAR)~fraction+viscosity+(1|ID), family =“binomial”), cbind(OCR,ECAR) = response variable; fraction, viscosity = factors; (1ID) = random factor SE = standard error. (TIF)

S4 Table Generalised linear mixed model(ATP production~fraction*viscosity+(1|ID), family =“nbinom”), SE = standard error; ATP production = response variable, fraction*viscosity = interaction between two factors, (1|ID) = random factor. (TIF)

S5 Table Generalised linear mixed model(ATP content~fraction+(1|ID), family =“nbinom”), SE = standard error; ATP content = response variable, fraction = factor, (1|ID) = random factor. (TIF)

S6 Table Linear mixed model (motility~(1|ID)), SE = standard error; df = degrees of freedom; motility = response variable, (1|ID) = random factor. (TIF)

S7 Table Linear mixed model (Tail length~fraction+(1|ID)), SE = standard error; df = degrees of freedom; tail length = response variable, fraction = factor, (1|ID) = random factor. (TIF)
