# Supplementary figures and images for: The motility-based swim-up technique separates bull sperm based on differences in metabolic rates and tail length
Source: PLoS One. 2019 Oct 10;14(10):e0223576. doi: 10.1371/journal.pone.0223576 (PMC6786571; doi:10.1371/journal.pone.0223576)

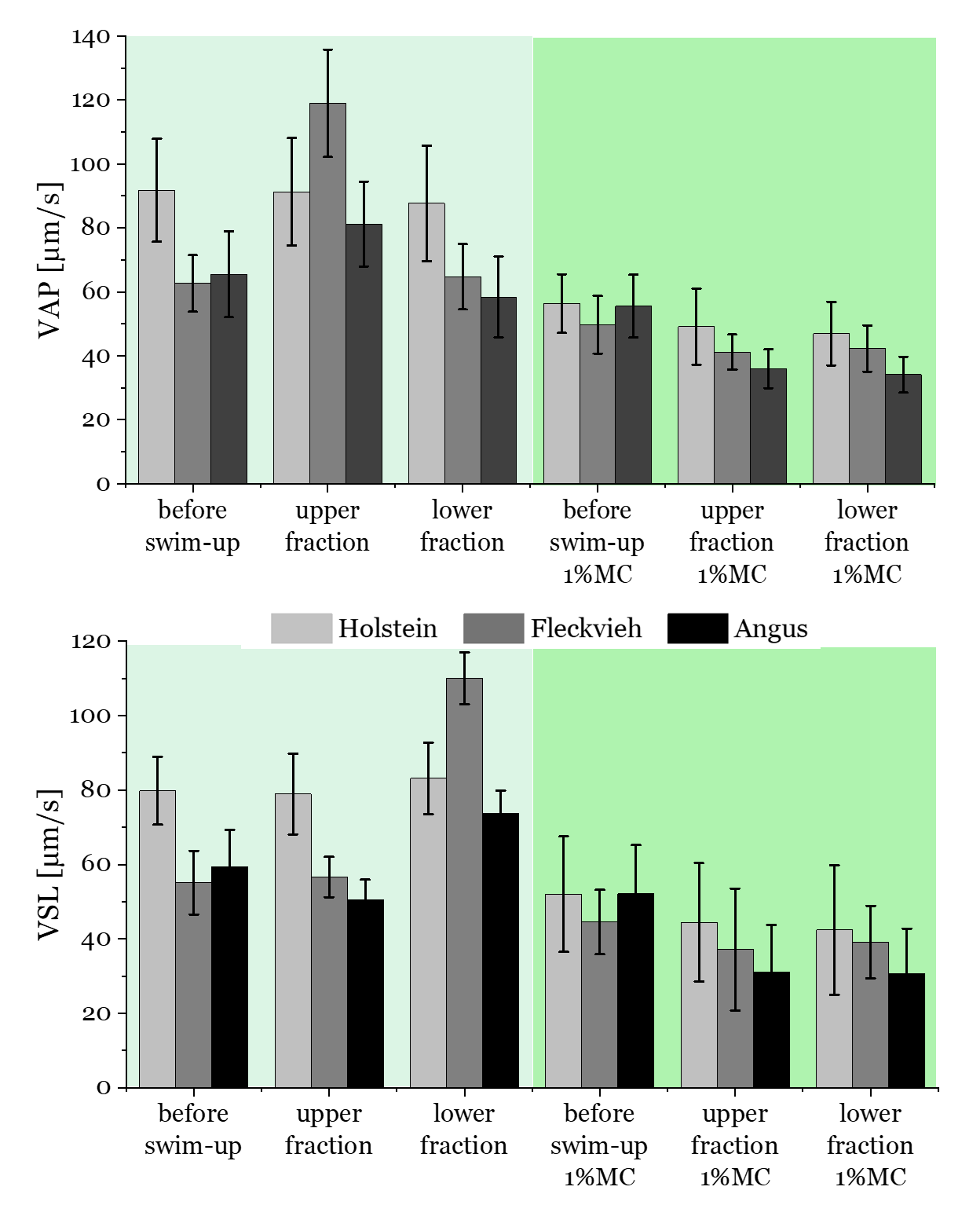

Supplement: S1 Fig — Average path velocity VAP (top) and straight line velocity VSL (bottom) of different bull sperm swim-up fractions in low viscosity (left) and high viscosity (right) for three different bulls. N >600 sperm cells for each bar. Videos of 10 second length were recorded and then analyzed in one second increments. For each condition and sperm fraction, 3 videos were recorded. Error bars show standard error of the mean. (TIF) [file pone.0223576.s001.tif]

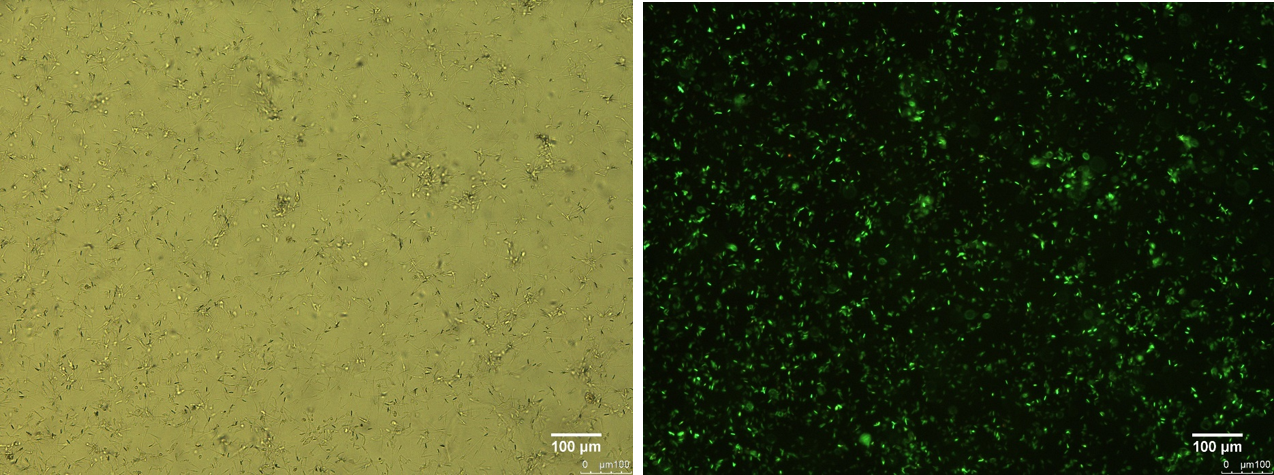

Supplement: S2 Fig — Sperm cell attachment (left image) during metabolic measurement in the XFp Analyzer and viability stain (right image) after seahorse measurement shows viable sperm cells under assay conditions (37°C, assay medium supplemented with glutamine and glucose). (TIF) [file pone.0223576.s002.tif]

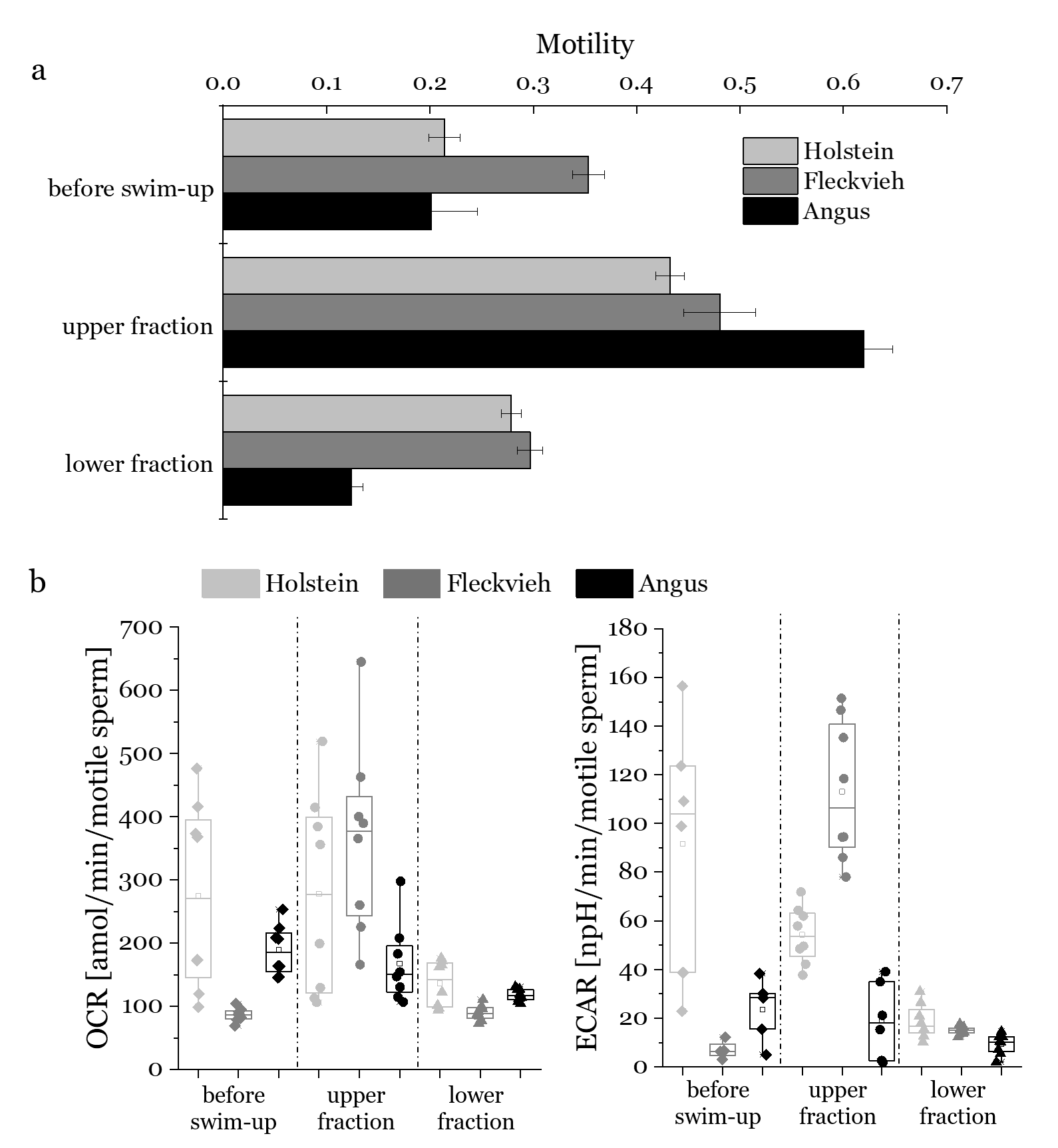

Supplement: S3 Fig — a) Overall motility of swim-up fractions of bull sperm. b) OCR (left) and ECAR (right) normalized to number of motile cells in each fraction. N≥4 replicate measurements. (TIF) [file pone.0223576.s003.tif]

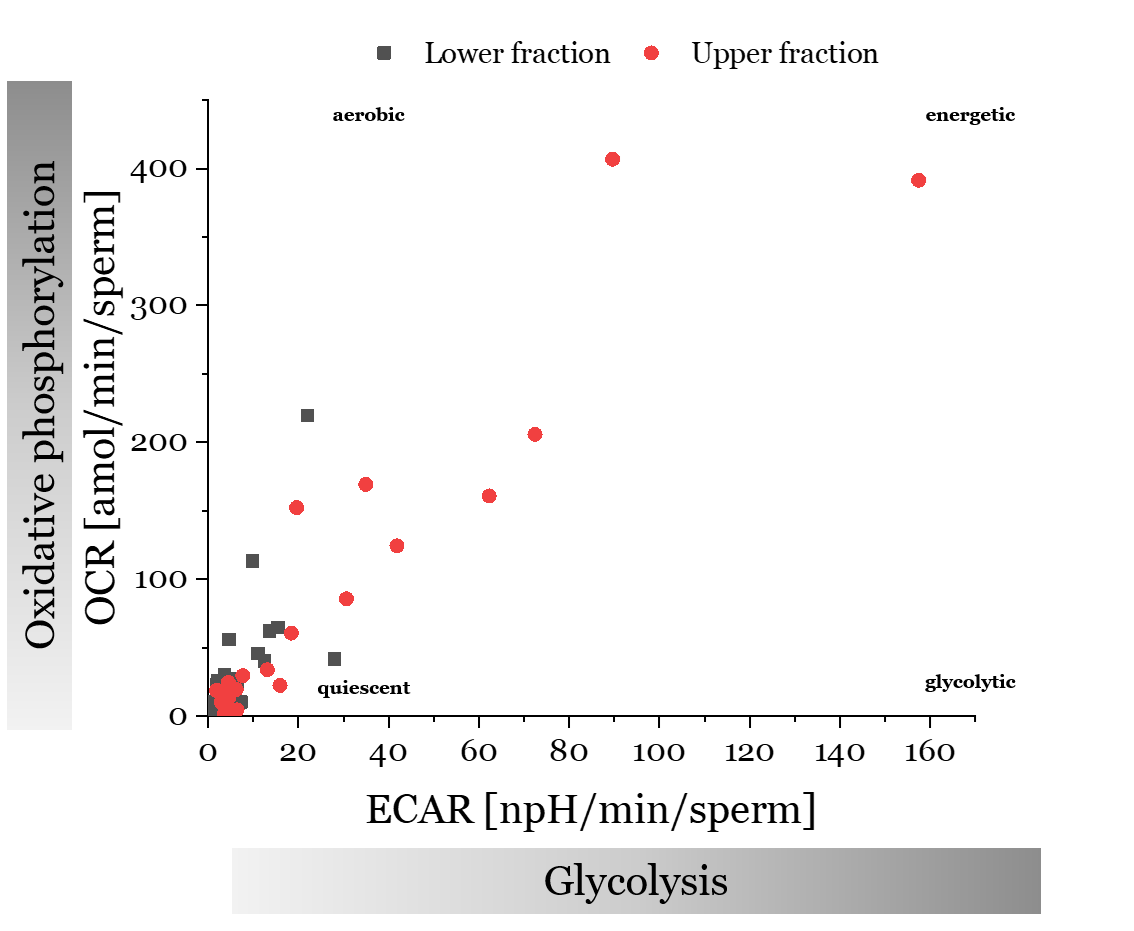

Supplement: S4 Fig — (TIF) [file pone.0223576.s004.tif]

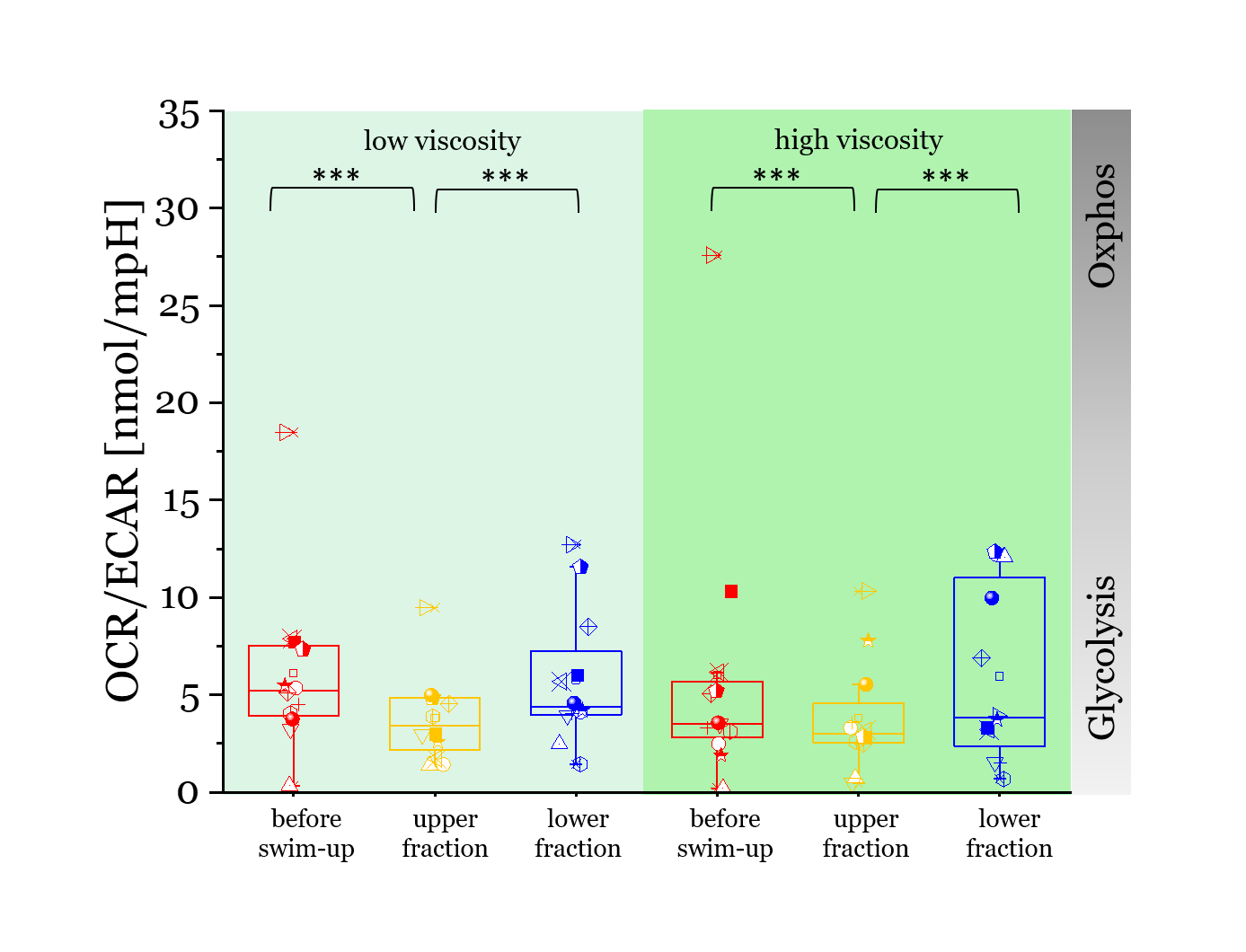

Supplement: S5 Fig — Each box displays OCR/ECAR ratios of 12 bulls, the horizontal line across each box is the median value. (TIF) [file pone.0223576.s005.tif]

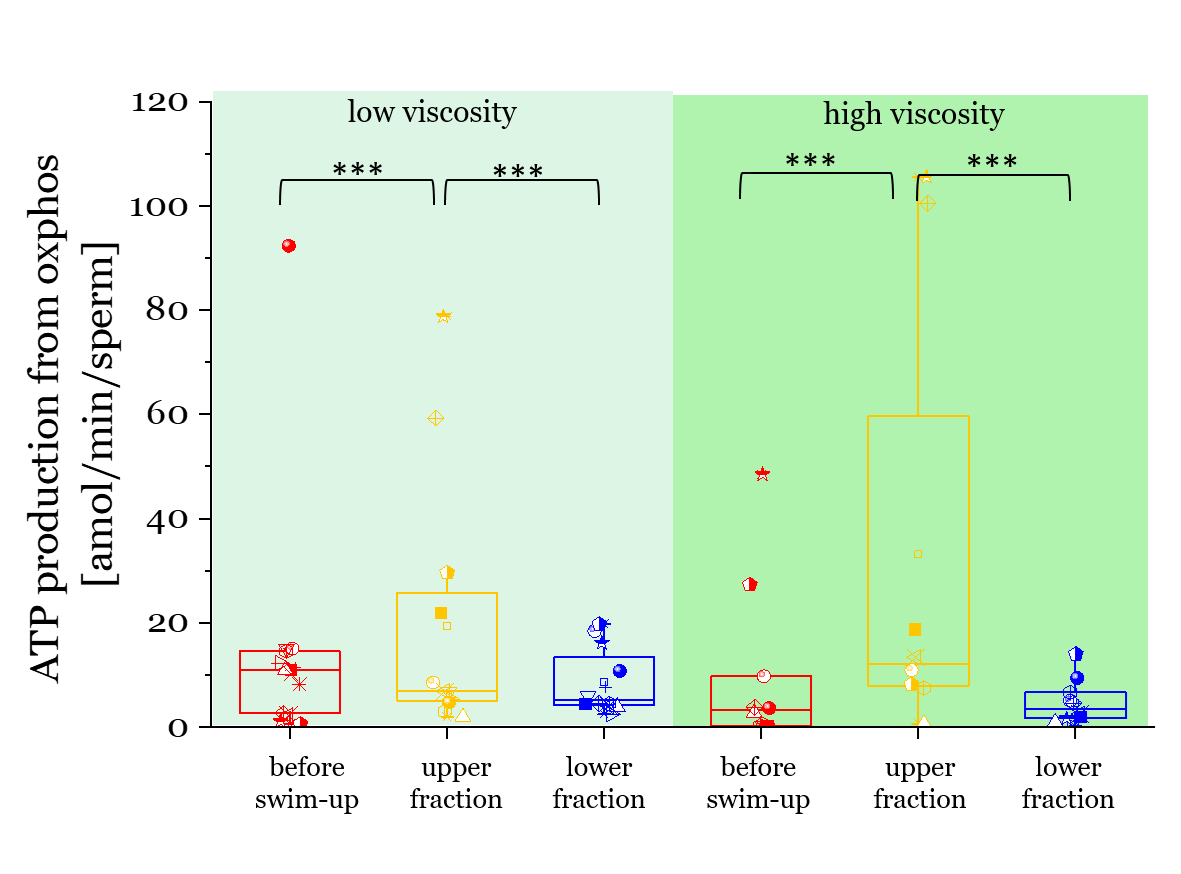

Supplement: S6 Fig — ATP production from oxphos calculated from oligomycin injections (see Fig 6A) of swim-up fractions in low viscosity (left panel) and high viscosity (right panel). Each box was obtained from 12 semen samples from different bulls. Horizontal lines through each box are the median values. Viscosity does not influence the ATP production significantly (p = 0.07). The ATP production in the upper fraction is significantly higher (p = 0.0001) than in the lower fraction and before swim-up. (TIF) [file pone.0223576.s006.tif]

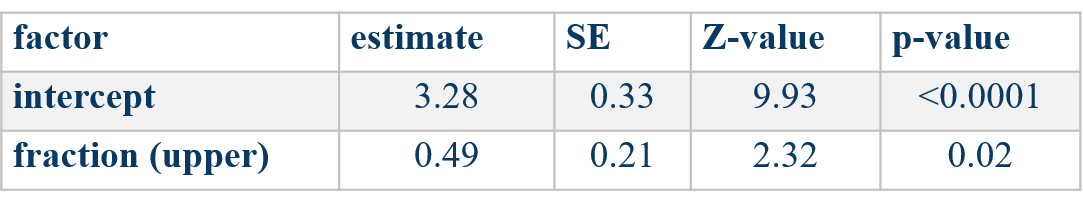

Supplement: S1 Table — OCR = response variable, fraction = factor, (1|ID) = random factor, SE = standard error. (TIF) [file pone.0223576.s007.tif]

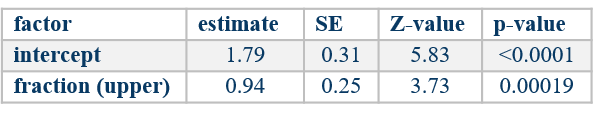

Supplement: S2 Table — (TIF) [file pone.0223576.s008.tif]

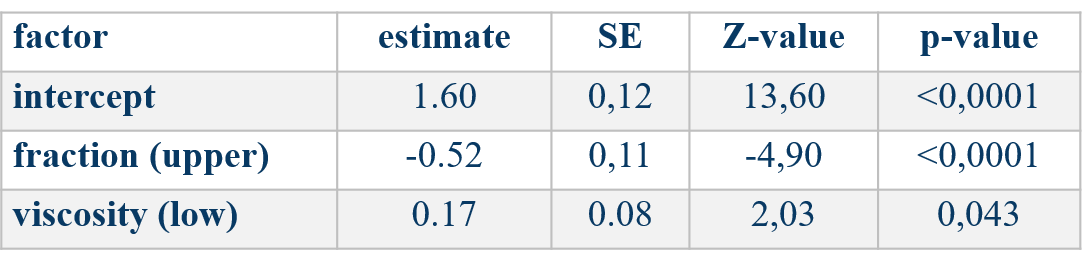

Supplement: S3 Table — (TIF) [file pone.0223576.s009.tif]

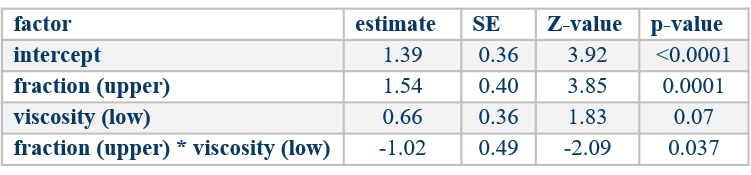

Supplement: S4 Table — (TIF) [file pone.0223576.s010.tif]

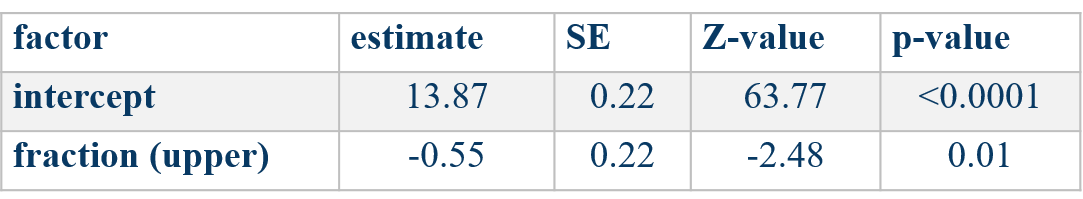

Supplement: S5 Table — (TIF) [file pone.0223576.s011.tif]

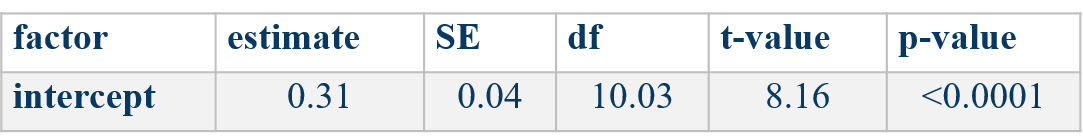

Supplement: S6 Table — (TIF) [file pone.0223576.s012.tif]

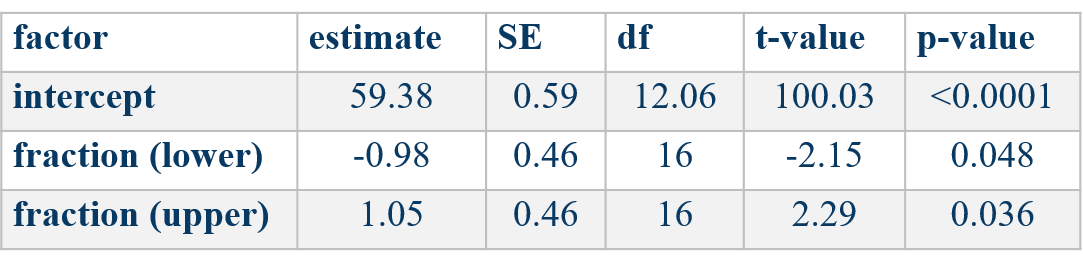

Supplement: S7 Table — (TIF) [file pone.0223576.s013.tif]
